# Neoadjuvant Immune Checkpoint Inhibitors Plus Chemotherapy in Locally Advanced Esophageal Squamous Cell Carcinoma: Perioperative and Survival Outcomes

**DOI:** 10.3389/fonc.2022.810898

**Published:** 2022-06-10

**Authors:** Xiao Ma, Weixin Zhao, Bin Li, Yongfu Yu, Yuan Ma, Mathew Thomas, Yawei Zhang, Jiaqing Xiang, Yiliang Zhang

**Affiliations:** ^1^ Department of Thoracic Surgery, Fudan University Shanghai Cancer Center, Institute of Thoracic Oncology, Fudan University, Shanghai, China; ^2^ Department of Oncology, Shanghai Medical College, Fudan University, Shanghai, China; ^3^ Department of Radiation Oncology, Fudan University Shanghai Cancer Center, Shanghai Key Laboratory of Radiation Oncology, Shanghai, China; ^4^ Department of Biostatistics, The Key Laboratory of Public Health Safety of Ministry of Education, School of Public Health, Fudan University, Shanghai, China; ^5^ Chinese Institute for Brain Research, Beijing, China; ^6^ Department of Cardiothoracic Surgery, Mayo Clinic, Jacksonville, FL, United States

**Keywords:** esophageal squamous cell carcinoma, neoadjuvant therapy, immune checkpoint inhibitor, esophagectomy, perioperative outcomes, survival outcomes

## Abstract

**Background:**

Immune checkpoint inhibitors (ICI) improve survival in patients with late-stage esophageal squamous cell carcinoma (ESCC) but have not been fully evaluated in locally advanced ESCC.

**Method:**

We retrospectively assessed outcomes of consecutive, treatment-naïve locally advanced ESCC (stage III or IVA) adults treated with neoadjuvant ICI plus chemotherapy followed by surgery, who refused or lacked access to radiotherapy, with regards to surgery feasibility, pathological response, and relapse-free survival (RFS).

**Results:**

We uneventfully treated 34 patients with the combined regimen in 2020. None reported grade III or higher toxic effects. All underwent surgery as planned: 32 received complete (R0) resections and 2 had microscopically positive margins (R1). Tumor downstaging occurred in 33 (97.1%) patients and 11 (32.4%) had pathologically complete response of the primary lesion. Median postoperative length of stay was 12 days (interquartile range: 11 to 17). All patients resumed a semi-liquid diet on discharge. The 90-day postoperative morbidity rate was 20.6% (7/34) with no mortalities. The 1-year RFS was 77.8% [95% CI, 64.2-94.2].

**Conclusion:**

Neoadjuvant ICI plus chemotherapy was safe and resulted in significant downstaging, rendering inoperable tumors operable, relieving symptoms of dysphagia and prolonging survival for locally advanced ESCC patients who refused or lacked access to radiotherapy.

## Introduction

Esophageal cancer is the sixth most common cause of cancer-related death worldwide and is therefore a major global health challenge (1). Esophageal squamous cell carcinoma (ESCC) is the main histologic type in East Asian and Middle Eastern countries. At the time of their first diagnosis, 40-50% of ESCC present as locally advanced esophageal cancer that invades local structures or involves regional lymph nodes but without distant metastases (2, 3). Surgery is recognized as the definitive treatment for this cancer, but the prognosis is poor with esophagectomy alone, mostly due to relapse of residual disease (4, 5). Neoadjuvant chemoradiotherapy followed by surgery, has shown promising survival benefit and been recommended as the standard management for resectable ESCC patients (6–9). However, radiotherapy has been reported to have a high risk of side effects that could preclude the planned surgical procedure (10–13). Moreover, it is not always available due to the lack of access to radiotherapy worldwide, especially in many low- and middle-income countries (14).

Compared to the standard strategy of preoperative chemoradiotherapy, the current neoadjuvant chemotherapy regimen without radiotherapy has significantly low disease-control rate and inferior histopathologic outcomes for locally advanced ESCC (12). Therefore, it is imperative to develop novel alternative treatment options for those who refuse or lack of access to radiotherapy. Immune checkpoint inhibitors (ICI), including both pembrolizumab and camrelizumab, combined with chemotherapy have recently been reported to be safe and effective in patients with late-stage ESCC (15, 16). One ICI, nivolumab, improved relapse-free survival (RFS) when used as adjuvant therapy in stage II/III resected esophageal cancer (17). This study was aimed to explore the preliminary outcomes of neoadjuvant ICI plus chemotherapy followed by surgery for patients with treatment-naïve, locally advanced ESCC.

## Patients and Methods

### Study Design

We performed this retrospective analysis of prospective collected data at a single medical institute. From January 1st, 2020 to December 31st, 2020, data of consecutive ESCC patients were prospectively collected in Fudan University Shanghai Cancer Center (FUSCC). The Institutional Review Board of FUSCC approved this study. All the patients provided written informed consents.

### Patient Eligibility

All patients underwent baseline tumor staging, including contrast-enhanced computed tomography (CT) of the chest and upper abdomen, ultrasound of the neck, and endoscopic ultrasound (EUS) of upper digestive tract with biopsy if necessary. Positron emission tomography (PET)/CT was suggested for those patients who could afford it as it was not covered by the common healthcare insurance yet (18–20).

Eligible patients were between 18 and 75 years of age, and had treatment-naïve ESCC located in the middle and lower thoracic esophagus and clinically staged as T3 to T4aN1 to N3 with no evidence of distant metastasis (M0) according to the American Joint Committee on Cancer (AJCC) 8th staging system (21). All the patients had an Eastern Cooperative Oncology Group (ECOG) performance status score of 0 or 1, adequate cardiopulmonary function, and no surgical contradictions. Key exclusion criteria were signs of esophageal perforation, immunodeficiency, ongoing systemic immunosuppressive therapy, active autoimmune or infectious disease, and clinically significant concurrent cancers.

### Treatment Protocol

The patients received two doses of intravenous pembrolizumab or camrelizumab (both at a dose of 200 mg every 3 weeks) plus chemotherapy with paclitaxel (260 mg/m^2^ every 3 weeks) and cisplatin (75 mg/m^2^ every 3 weeks) for 2 cycles. Surgery was planned to be performed within 14 weeks after the last dose if the patients met the following surgical criteria: 1) the tumor was considered to completely resectable upon evaluation of the multidisciplinary team; 2) the patient had the physiological conditions for upper gastrointestinal reconstruction after esophagectomy; 3) there’s no contraindications to general anesthesia; 4) the patient refused radiation therapy. The primary end points were surgery feasibility rate, including the proportion of patients able to undergo surgery after neoadjuvant therapy, completeness of resection, and 90-day post-operative morbidity and mortality rates. The secondary end points were pathologic response and RFS rates. Drug toxicities were assessed according to the Common Terminology Criteria for Adverse Events (CTCAE v5.0). Changes in tumor size were evaluated according to Response Evaluation Criteria in Solid Tumors (RECIST), version 1.1. Surgical procedures of esophagectomy and lymph node dissection were conducted according to FUSCC institutional standards (22–25). The complications were specified and evaluated based on the International Consensus on Standardization of Data Collection for Complications Associated with Esophagectomy (26) and the Clavien-Dindo classification of surgical complications (27).

### Pathological Assessment

Surgical specimens were assessed and staged according to the AJCC 8th criteria for evaluating tumor size, invasion depth, resection margin, and affected lymph nodes, for the percentage of residual viable tumor that was identified on routine hematoxylin and eosin staining (21). Pathologic response was evaluated and classified using the internationally recognized standards of tumor regression grade (TRG) system, based on two parameters of histomorphologic tumor regression and lymph node status (ypN) (28). Those with no evidence of vital residual tumor cells in both primary tumor and lymph nodes were considered to have pathological complete response (pCR).

### Statistical Analysis

The patients were characterized by demographic and clinicopathologic variables. Differences in patient features were evaluated using chi-square tests for categorical variables and Wilcoxon rank-sum tests for continuous variables. All statistical analyses were two-sided, with p < 0.05 indicative of statistical significance, and performed using SPSS (version 22.0 IBM Corporation, Armonk, NY) and R 4.0.3 software (R Foundation for Statistical Computing, Vienna, Austria).

## Results

### Patient Characteristics

Thirty-four patients underwent treatment with the combined protocol during the 1-year study period (**Table 1**). They had either stage III (41.2%) or IVA (58.8%) ESCC and received two cycles of chemotherapy plus pembrolizumab (n=20) or camrelizumab (n=14). The median age of these patients was 61 years (range: 47-74). The majority of the cohort consisted of males (91.2%) and smokers (59%). Nearly half of the patients (47.1%) were documented to have a history of alcohol addiction. On initial evaluation by the thoracic surgeons, all patients were considered to have tumors that could not be completely resected.

**Table 1:** Baseline demographics and clinical characteristics.

Variables (%)	All Patients (N=34)	Pembrolizumab (N=20)	Camrelizumab (N=14)
**Sex**			
** Female**	3 (8.8%)	0	3 (21.4%)
** Male**	31 (91.2%)	20 (100%)	11 (78.6%)
**Age**			
** Median (range)**	61 (47-74)	60.5 (47-74)	63 (55-68)
**ECOG**			
** 0**	25 (73.5%)	15 (75%)	10 (71.4%)
** 1**	9 (26.5%)	5 (25%)	4 (28.6%)
**BMI, kg/m^2^**			
** Median (range)**	22.2 (14.7-29.8)	21.6 (14.7-26.0)	22.4 (17.5-29.8)
**Smoking**	20 (59%)	14 (70%)	6 (43%)
**Alcohol addiction**	16 (47.1)	12 (60%)	4 (28.5%)
**Tumor location**			
** Middle**	25 (73.5%)	13 (65%)	12 (85.7%)
** Lower**	9 (26.5%)	7 (35%)	2 (14.3%)
**Clinical T stage**			
** cT3**	26 (76.5%)	15 (75%)	11 (78.6%)
** cT4a**	8 (23.5%)	5 (25%)	3 (25%)
**Clinical N stage**			
** cN2**	16 (47%)	9 (45%)	7 (50%)
** cN3**	18 (53%)	11 (55%)	7 (50%)
**Clinical stage**			
** III**	14 (41.2)	8 (40%)	6 (43%)
** IVA**	20 (58.8)	12 (60%)	8 (57%)
**Grade**			
** G1**	1 (3%)	1 (5%)	0
** G2**	13 (38.2%)	9 (45%)	4 (28.6%)
** G3**	9 (26.5%)	4 (20%)	5 (35.7%)
** GX**	11 (32.3%)	6 (30%)	5 (35.7%)

### Safety and Feasibility

Therapy-related adverse events of any grade during the neoadjuvant regimen occurred in 58.8% (20/34) patients, but none were grade III or higher. The most common adverse incidents were grade 1 digestive tract-associated side effects (8/34), such as nausea, vomiting and diarrhea. Another adverse event of high incidence was reactive capillary endothelial proliferation (7/34; grade 1 in 6 patients and grade 2 in one patient), which was commonly associated with camrelizumab (16). The median interval between the administration of the second dose and surgery was 5 weeks (range 4-8 weeks), and there were no therapy-related surgical delays. FUSCC multidisciplinary team for thoracic cancer evaluated the medical data of each patient including the symptoms, endoscopic and radiological findings. All patients showed partial response (PR) or stable disease (SD) and underwent surgery with intent to curative treatment; 16 (47%) received esophagectomy with 2-field lymphadenectomy whereas 18 (53%) had 3-field lymphadenectomy. Regarding the anastomotic site, half of the patients underwent intra-thoracic anastomosis and half cervical procedure. The average operating time was 211 ± 47 min, and the intraoperative blood loss was 144 ± 126 ml. There were 7 (20.6%) patients who experienced postoperative complications, which were all below grade IIIa according to Clavien-Dindo classification. No patients died within 90 days after surgery. All patients resumed a semi-liquid diet at the time of discharge which relieved their chief complaint of dysphagia noted at the initial clinic visit. (**Tables 2**, **3**).

**Table 2:** Adverse events during neoadjuvant immune checkpoint inhibitors plus chemotherapy and postoperative complications.

Events (%)	All patients (N=34, %)	Grade	Pembrolizumab (N=20)	Camrelizumab (N=14)
**All events during neoadjuvant therapy**	20 (58.8%)	/	6 (30%)	14 (100%)
Nausea/vomiting/diarrhea	8	I	4	4
Reactive capillary endothelial proliferation	7	I/II	0	7
Fatigue	3	I	1	2
Leukopenia	2	I	1	1
**All postoperative complications**	7 (20.6%)	/	5 (25%)	2 (14.3%)
Anastomotic leak	3	II	2	1
Pneumonia	2	II	2	0
hoarseness	1	I	1	0
Subcutaneous emphysema	1	I	0	1
**90-day Postoperative mortality**	0	/	0	0

Drugs toxicity was assessed and graded according to the Common Terminology Criteria for Adverse Events (CTCAE v5.0). Postoperative complications were evaluated by the Clavien-Dindo classification.

**Table 3:** Surgery, pathologic response and survival outcomes.

Variables (%)	All Patients (N=34)	Pembrolizumab (N=20)	Camrelizumab (N=14)
**Operative duration, mean (SD), min**	211 (47)	204 (40)	222 (55)
**Estimated blood loss, mean (SD), mL**	144 (126)	144 (126)	144 (127)
**Postoperative hospital stay, median (IQR), day**	12 (11-17)	13 (11-23)	12 (10-13)
**Completeness of Resection**			
** R0**	32 (94.1%)	19 (95%)	13 (92.9%)
** R1**	2 (5.9%)	1 (5%)	1 (7.1%)
**Lymph nodes resected, median (IQR), No**	30 (25-38)	31 (25-37)	27 (23-37)
**Complete response of primary tumor**	11 (32.4%)	6 (30%)	5 (35.7%)
**TRG1**	8 (23.4)	4 (20)	4 (28.6)
**TRG**			
** 1**	8 (23.5%)	4 (20%)	4 (28.6%)
** 2**	3 (8.8%)	2 (10%)	1 (7.1%)
** 3**	7 (20.6)	5 (25%)	2 (14.3%)
** 4**	16 (47.1%)	9 (45%)	7 (50%)
**ypT**			
** 0**	11 (32.4%)	6 (30%)	5 (35.7%)
** 1**	6 (17.6%)	5 (25%)	1 (7.2%)
** 2**	5 (14.7%)	3 (15%)	2 (14.2%)
** 3**	12 (35.3%)	6 (30)	6 (42.9%)
**ypN+**	19 (55.9%)	11 (55%)	8 (57%)
**ypStage**			
** I**	13 (38.2%)	7 (35%)	6 (42.9%)
** II**	2 (5.9%)	5 2(10%)	0
** III**	12 (35.3%)	6 (30%)	6 (42.9)
** IV**	7 (20.6%)	5 (25%)	2 (14.2%)
**1-year relapse events**	7 (20.6%)	5 (25%)	2 (14.2%)
**1-year RFS,% (95%CI)**	77.8 (64.2-94.2)	72.7 (54.5-97)	85.7 (69.2-100)

IQR, interquartile range; pCR, pathological complete response; defined as no evidence of residual viable tumor cells in the resected primary tumor and lymph nodes. TRG, tumor regression grade, based on two parameters of histomorphologic tumor regression and lymph node status (ypN) (28). RFS, relapse-free survival.

### Radiologic and Pathologic Response

**Figure 1:**
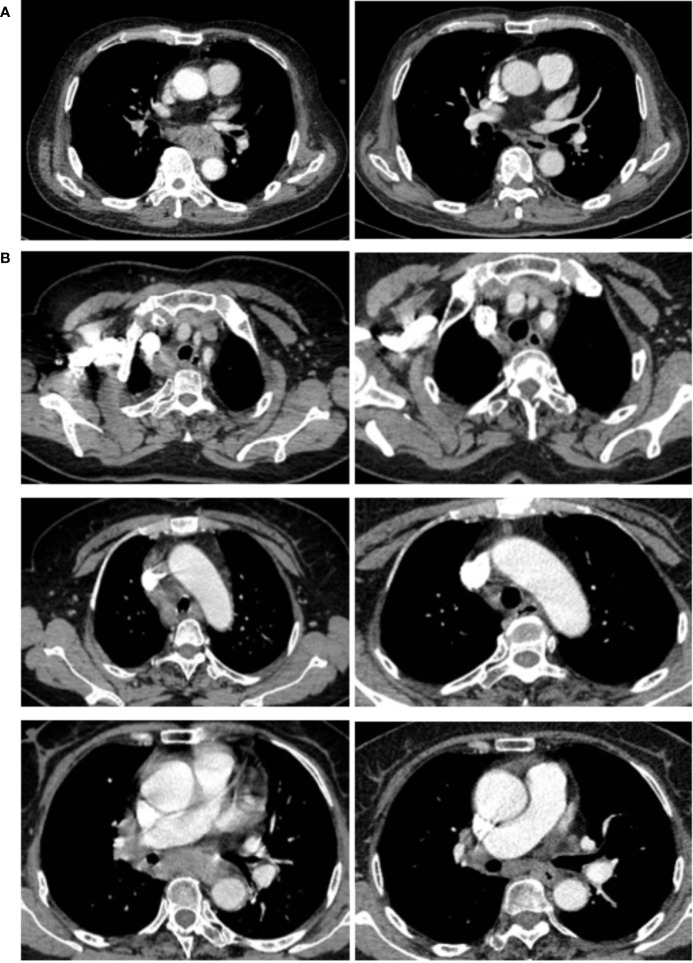
Cases of radiological responses after neoadjuvant immune checkpoint inhibitors and chemotherapy. **(A)** This shows the radiological images of a 67-year-old male (patient 1) with a stage IVA ESCC before and after neoadjuvant treatment. This patient achieved pathological regression of 100% for esophageal lesion with no residual lymph node metastasis according to postoperative specimen **(B)** This shows the images of a 68-year-old female (patient 2), who had a stage IVA ESCC before neoadjuvant treatment. This patient had 100% pathological regression of the primary tumor but had residual metastatic lymph nodes.

Representative radiologic responses after two preoperative doses of ICI plus chemotherapy are shown in **Figure 1**. The evaluation and comparison of the radiographic results before and after the neoadjuvant therapy were described previously (29). Surgical pathology revealed 32 (94.1%) patients had complete resection (R0) of the primary tumor and the local lymph nodes, while 2 (5.9%) had macroscopic negative resection but positive circumferential margins microscopically (R1) on the resected esophagus. The median number of resected lymph nodes for each patient was 30 (interquartile range (IQR): 25 to 38). Based on the 8th AJCC system for clinical and pathologic staging, all patients were down-staged after neoadjuvant therapy; 28 (82.3%) patients had significant tumor (T) shrinkage, and 27 (79.4%) had nodal (N) downstaging. Tumor regression grade (TRG) I, II, III and IV, defined by two parameters of histomorphologic tumor regression and lymph node status (ypN) (28), were observed in 23.5%, 8.8%, 20.6% and 47.1% of patients, respectively. Complete pathologic response of the primary tumor site (ypT0) was seen in 11 (32.4%) patients, but 3 of them had residual cancer cells in the resected lymph nodes (**Table 3** and **Supplementary Table**).

### Survival Outcomes

**Figure 2:**
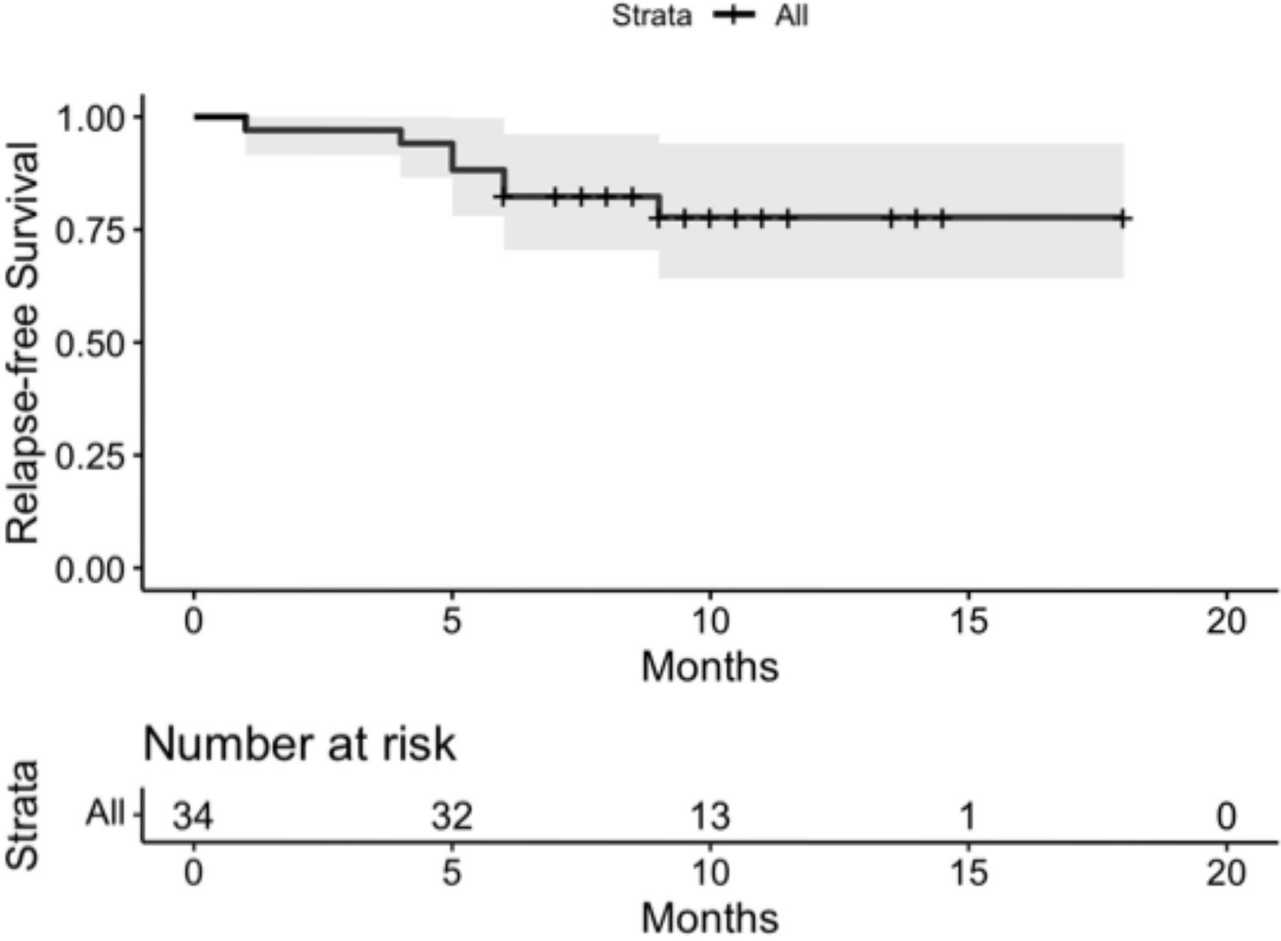
For the entire cohort, the 1-year Relapse-free survival (RFS) was 77.8% (95% confidential interval (CI) 64.2% to 94.2%).

The median follow-up time was 9.5 months (IQR: 8.5 to 11 months. 7 patients had documented disease recurrence, of whom 3 developed supraclavicular lymph node metastases and 4 distant spread, including bone, brain and liver involvement. At the time of their most recent follow up, no deaths occurred as a result of esophageal cancer; one patient died while undergoing treatment for primary kidney cancer. For the entire cohort, the 1-year RFS was 77.8% (95% confidential interval (CI) 64.2% to 94.2%) (**Figure 2**). No significant difference in survival was observed between the 2 ICI drugs of pembrolizumab and camrelizumab. Overall survival data were not mature (**Table 3**).

## Discussion

Our study shows that two cycles of preoperative ICI plus chemotherapy were well tolerated in locally-advanced ESCC patients, without therapy-related surgical delays. Furthermore, the preoperative regimen provided significant disease downstaging, turning unresectable ESCC into completely resectable tumors. More importantly, our data showed the introduction of preoperative ICI drugs did not increase the surgical difficulty or the postoperative complication, including treatment-related mortality. On the short-term follow-up, our study cohort demonstrated favorable 1-year RFS without any deaths from the late-stage disease. Therefore, this preoperative strategy allowed locally advanced ESCC that were unlikely to be surgical candidates at the first diagnosis to be completely removed eventually, without the need for radiotherapy. In this way, the novel treatment method could relieve dysphagia symptom of these patients, but also potentially extend their long-term survival.

Several clinical trials are currently evaluating the neoadjuvant role of ICI combined with chemoradiotherapy for esophageal cancer (NCT03604991, NCT03087864, NCT03044613, NCT02844075 and NCT03792347), some of which have reported preliminary outcomes confirming the high degree of safety and feasibility of the treatment strategy (30). Our previous work demonstrated that the use of neoadjuvant ICI plus chemotherapy could achieve a rate of over 40% of major pathologic response (MPR) in ESCC, without increasing the complication rates during the therapy and surgery (29).

Our study also brings special attention to a particular dilemma regarding treatment response. We noted that in 8.8% (3/34) of patients, although the primary site had pCR following neoadjuvant therapy, persistent disease was still present within involved lymph nodes. A similar observation has also been made by other investigators. PALACE-1 which is a phase II multicenter study aiming to evaluate preoperative pembrolizumab combined with chemoradiotherapy for resectable ESCC recently published its preliminary results, showing 11% (2/18) achieved pCR in primary tumor, however, had residual cancer cells in resected lymph nodes (30). The management of these patients with persistent nodal disease has opened up a new challenge for oncologists. It is unclear whether there is any benefit for adjuvant therapy using the original regimen after complete resection of ESCC. On the one hand, these patients who harbored residual cancer cells in the lymph nodes showed definite pathologic proof that their tumor was well responsive to ICI plus chemotherapy. On the other, current guidelines suggest that there is no proven benefit of adjuvant therapy for ESCC (3, 31). CheckMate 577 (17), a global, randomized, double-blind, placebo-controlled phase 3 trial to evaluate adjuvant therapy with ICI in esophageal or gastroesophageal junction cancer, reported that among patients who underwent resection after neoadjuvant chemoradiotherapy, RFS was significantly longer with nivolumab adjuvant therapy compared to placebo therapy. It should be noted that the majority (71%) of CheckMate 577 participants had adenocarcinoma and only 29% ESCC. We anticipate that studies focusing on the ICI adjuvant therapy for ESCC patients will provide more data, and could possibly result in new guidelines.

Our study has certain limitations that should be addressed. First, although this study may have the largest sample size of ESCC patients receiving neoadjuvant ICI plus chemotherapy followed by surgery to date, it is likely that some sort of selection bias was present due to the nature of monocentric series in a single institution. The inclusion of a validation cohort would have strengthened the findings of the study. Second, the study population was limited to East Asians, thereby raising concerns about the generalizability of our results, since disease spectra as well as biological and pathologic characteristics may differ among ethnic groups. Third, this is a retrospective single-armed study, and the results should be further validated by multi-institutional prospective randomized-controlled trials. Encouragingly, there are several ongoing prospective clinical trials which will provide more evidence in this area.

In summary, neoadjuvant ICI plus chemotherapy was safe for those patients with locally advanced ESCC who refused or lacked access to radiotherapy. This treatment regimen provided significant tumor downstaging rendering inoperable tumors operable, relieving symptoms of dysphagia and prolonging survival.

## Data Availability Statement

The original contributions presented in the study are included in the article/**Supplementary Material**. Further inquiries can be directed to the corresponding authors.

## Author Contributions

JX and YiZ conceptualized and designed the study; XM and WZ collected data, follow-up, wrote the manuscript; BL, YY and YM performed data cleansing and statistical analysis; BL, YaZ, JX and YiZ participated in the surgery and perioperative management. MT and YiZ established the clinical management protocols and edited the manuscript. All authors have reviewed, discussed, and approved the manuscript.

## Funding

This work is supported by Shanghai Pujiang Program (2020PJD014).

## Conflict of Interest

Author MT was employed by Mayo Clinic.

The remaining authors declare that the research was conducted in the absence of any commercial or financial relationships that could be construed as a potential conflict of interest.

## Publisher’s Note

All claims expressed in this article are solely those of the authors and do not necessarily represent those of their affiliated organizations, or those of the publisher, the editors and the reviewers. Any product that may be evaluated in this article, or claim that may be made by its manufacturer, is not guaranteed or endorsed by the publisher.

## Abbreviations

AJCC, American Joint Committee on Cancer; CI, Confidential interval; CT, Computed tomography; CTCAE, Common Terminology Criteria for Adverse Events; ECOG, Eastern Cooperative Oncology Group; ESCC, Esophageal squamous cell carcinoma; EUS, Endoscopic ultrasound; FUSCC, Fudan University Shanghai Cancer Center; HR, Hazard ratio; ICI, Immune checkpoint inhibitors; pCR, pathologic complete response; PET, Positron emission tomography; RECIST, Response Evaluation Criteria in Solid Tumors; RFS, Relapse-free survival; TNM, Tumor Nodes Metastases; TRG, Tumor regression grade.
