# Supplementary material for: Neoadjuvant Immune Checkpoint Inhibitors Plus Chemotherapy in Locally Advanced Esophageal Squamous Cell Carcinoma: Perioperative and Survival Outcomes
Source: Front Oncol. 2022 Jun 10;12:810898. doi: 10.3389/fonc.2022.810898 (PMC9226664; doi:10.3389/fonc.2022.810898)
Supplement: Supplementary file 1 [file Table_1.docx]

We made an independent table listing the tumor downstaging information. (please see table)

| Patents | cT | cN | cM | cTNM | ypT | ypN | ypM | ypStage |
| --- | --- | --- | --- | --- | --- | --- | --- | --- |
| 1 | 4a | 2 | 0 | IVA | 0 | 3 | 0 | IVA |
| 2 | 3 | 3 | 0 | IVA | 0 | 3 | 0 | IVA |
| 3 | 3 | 3 | 0 | IVA | 1b | 3 | 0 | IVA |
| 4 | 3 | 3 | 0 | IVA | 2 | 3 | 0 | IVA |
| 5 | 4a | 3 | 0 | IVA | 3 | 3 | 0 | IVA |
| 6 | 4a | 3 | 0 | IVA | 3 | 3 | 0 | IVA |
| 7 | 4a | 3 | 0 | IVA | 3 | 3 | 0 | IVA |
| 8 | 3 | 2 | 0 | III | 0 | 0 | 0 | I |
| 9 | 3 | 2 | 0 | III | 0 | 0 | 0 | I |
| 10 | 3 | 2 | 0 | III | 0 | 0 | 0 | I |
| 11 | 3 | 2 | 0 | III | 0 | 0 | 0 | I |
| 12 | 3 | 2 | 0 | III | 0 | 0 | 0 | I |
| 13 | 3 | 2 | 0 | III | 1b | 0 | 0 | I |
| 14 | 3 | 2 | 0 | III | 2 | 0 | 0 | I |
| 15 | 3 | 2 | 0 | III | 2 | 0 | 0 | I |
| 16 | 3 | 2 | 0 | III | 0 | 0 | 0 | I |
| 17 | 3 | 3 | 0 | IVA | 0 | 0 | 0 | I |
| 18 | 3 | 3 | 0 | IVA | 0 | 0 | 0 | I |
| 19 | 4a | 3 | 0 | IVA | 1a | 0 | 0 | I |
| 20 | 3 | 3 | 0 | IVA | 1b | 0 | 0 | I |
| 21 | 3 | 2 | 0 | III | 3 | 0 | 0 | **II** |
| 22 | 3 | 2 | 0 | III | 3 | 0 | 0 | II |
| 23 | 3 | 2 | 0 | III | 1b | 1 | 0 | IIIA |
| 24 | 3 | 2 | 0 | III | 1b | 1 | 0 | IIIA |
| 25 | 3 | 3 | 0 | IVA | 0 | 1 | 0 | IIIA |
| 26 | 3 | 3 | 0 | IVA | 2 | 1 | 0 | IIIA |
| 27 | 3 | 2 | 0 | III | 3 | 1 | 0 | IIIB |
| 28 | 4a | 2 | 0 | III | 3 | 1 | 0 | IIIB |
| 29 | 3 | 3 | 0 | IVA | 2 | 2 | 0 | IIIB |
| 30 | 4a | 3 | 0 | IVA | 3 | 1 | 0 | IIIB |
| 31 | 3 | 3 | 0 | IVA | 3 | 2 | 0 | IIIB |
| 32 | 3 | 3 | 0 | IVA | 3 | 2 | 0 | IIIB |
| 33 | 3 | 3 | 0 | IVA | 3 | 2 | 0 | IIIB |
| 34 | 4a | 2 | 0 | IVA | 3 | 1 | 0 | IIIB |
